# Preparation and Properties of C/C Hollow Spheres and the Energy Absorption Capacity of the Corresponding Aluminum Syntactic Foams

**DOI:** 10.3390/ma11060997

**Published:** 2018-06-12

**Authors:** Qiyong Yu, Yan Zhao, Anqi Dong, Ye Li

**Affiliations:** School of Materials Science and Engineering, Beihang University, Beijing 100191, China; yqy_318@163.com (Q.Y.); dong20121226@gmail.com (A.D.); zhuliye1101@126.com (Y.L.)

**Keywords:** hollow carbon spheres, syntactic foam, rolling ball, preparation

## Abstract

The present study focuses on the preparation and characterization of lab-scale aluminum syntactic foams (ASFs) filled with hollow carbon spheres (HCSs). A new and original process for the fabrication of HCSs was explored. Firstly, expanded polystyrene beads with an average diameter of 6 mm and coated with carbon fibers/thermoset phenolic resin were produced by the “rolling ball” method. In the next step, the spheres were cured and post-cured, and then carbonized at 1050 °C under vacuum to form the HCSs. The porosity in the shell of the HCSs was decreased by increasing the number of impregnation–carbonization cycles. The aluminum syntactic foams were fabricated by casting the molten aluminum into a crucible filled with HCSs. The morphology of the hollow spheres before and after carbonization was investigated by scanning electron microscope (SEM). The compressive properties of the ASF were tested and the energy absorption capacities were calculated according to stress–strain curves. The results showed that the ASF filled with HCSs which had been treated by more cycles of impregnation–carbonization had higher energy absorption capacity. The aluminum syntactic foam absorbed 34.9 MJ/m^3^ (28.8 KJ/Kg) at 60% strain, which was much higher than traditional closed cell aluminum foams without particles. The HCSs have a promising future in producing a novel family of metal matrix syntactic foams.

## 1. Introduction

Metallic syntactic foams are synthesized by dispersing rigid hollow particles in a metal matrix material. Metallic syntactic foams are endowed with good energy absorption capacity and therefore carry great potential in packing, automobile, aerospace, and construction industries [1]. The mechanical properties of syntactic foams depend on both filler and matrix properties [2,3]. Hollow ceramic microspheres are the most popular filler used in producing metal matrix syntactic foams due to the excellent properties of low density and high temperature resistance. However, most of the ceramic microspheres have a density range of 0.7–0.9 g/cm^3^, which is relatively high for syntactic foams. Ceramic microspheres of small size are difficult to infiltrate with molten metal. A high pressure gas assisted infiltration technique must be used. However, the hollow spheres may be cracked prior to or during infiltration because of the high pressure applied on the spheres [4]. 

The carbon fiber/carbon matrix (C/C) composites have plenty of advantages, such as low density, high strength, high specific modulus, high thermal conductivity, low thermal expansion coefficient, good thermal shock resistance, and high dimensional stability [5,6]. The C/C composites are considered to be one of the most promising high temperature resistant materials, able theoretically to withstand temperatures up to 2600 °C [7,8]. Based on these properties, the hollow C/C spheres are one of the most appropriate materials to be used as the filler in metal matrix syntactic foams. Carbonization of core–shell polymer particles is an effective method to fabricate hollow carbon spheres. The core–shell polymer spheres, which are synthesized using expanded polystyrene (EPS) beads as sacrificial templates, have attracted considerable attention [9,10,11]. The preparation mechanism was such that the core material of EPS had a low decomposition temperature, while the shell polymer had a high decomposition temperature. The shell polymer maintained its shape well after the shrinkage of the EPS.

The volume fraction of the hollow particles is one of the key factors affecting the properties of metal matrix syntactic foams: density, coefficient of thermal expansion, compressive strength, etc. According to the published literature, the volume fraction in random arrays of identical spheres varies from 0.59 to 0.64, depending on packing conditions. A simple and easy way to increase particle volume fractions is to pack particles having different sizes [12,13].

Compared with hollow microspheres, it is more difficult to produce hollow macrospheres that have the properties of high temperature resistance and high compressive strength. Macrospheres have lower threshold pressure for initiation of the infiltration process by liquid aluminum compared with microspheres [14].

In this work, a new type of macro-hollow carbon spheres (HCSs) was prepared by carbonization of EPS/phenolic core–shell polymer particles. The HCSs were then used as fillers to fabricate aluminum syntactic foam (ASF).In order to improve compressive property of the HCSs and the corresponding syntactic foams, the HCSs with different impregnation–carbonization cycles were produced and tested. The cross-sectional area of HCSs was investigated by scanning electron microscope (SEM). The density and energy absorption capacity of the ASF were also tested and studied.

## 2. Materials and Methods

### 2.1. Materials

Four basic materials including carbon fibers, phenolic resin, EPS beads, and aluminum alloy were used. The average length of the carbon fibers obtained from Toray Industries(Tokyo, Japan) was approximately 300 μm. A thermoset phenolic resin with a density of 1.25 g/cm^3^ was used as carbon precursor with carbon yield percentage of about 65%. The EPS beads (Ø6 mm, 10 kg/m^3^) were a light ball made by expandable polystyrene through chemical foaming, which were used as initiation template. We chose the cast Al-Si alloy ZL101 as the matrix material, as it has excellent castability and good mechanical properties. The main chemical components had a silicon content of 6.5–7.5%, and Mg content of 0.2–0.4%, and a melting range of 557~613 °C. The equipment used in this paper include a “rolling ball” coater machine and a vacuum carbonization furnace.

### 2.2. Test Method

Quasi-static compression testing of the ASFs was performed on an Instron 5667 universal testing machine (Instron Corporation, Norwood, MA, USA) with a 10 kN load cell according to ISO13314-2011. Testing was carried out at a constant cross head speed of1.0 mm/min at room temperature. In order to avoid size effects, the diameter of the sample should be at least 7–10 times that of pore size. The dimension of the specimen used in this paper was Ø45 mm× 55 mm. Five specimens for compressive testing were prepared in each group. The density of the hollow spheres was measured by the Archimedes method in accordance with ASTM D792-13. The spheres were firstly weighed in air. They were then immersed in a measuring cup filled with pure water, their apparent mass upon immersion was determined, and their specific gravity (relative density) was calculated.

The micro-structure of the carbon fiber/phenolic resin before and after the carbonization was observed by a scanning electron microscope operating in low-vacuum mode (Jeol JSM 5410LV, Tokyo, Japan). 

The cross-section morphology of ZL101 was also investigated by SEM. Sections were cut from the ASF for standard grinding and polishing. Samples were ground using 180-, 240-, 320-, 600-, and 1200-grit silicon carbide papers. A mirror-like surface finish was achieved by subsequent polishing with 0.5  μm and 0.05 μm diamond powder suspended in distilled water.

### 2.3. Preparation of HCSs

**Figure 1:**
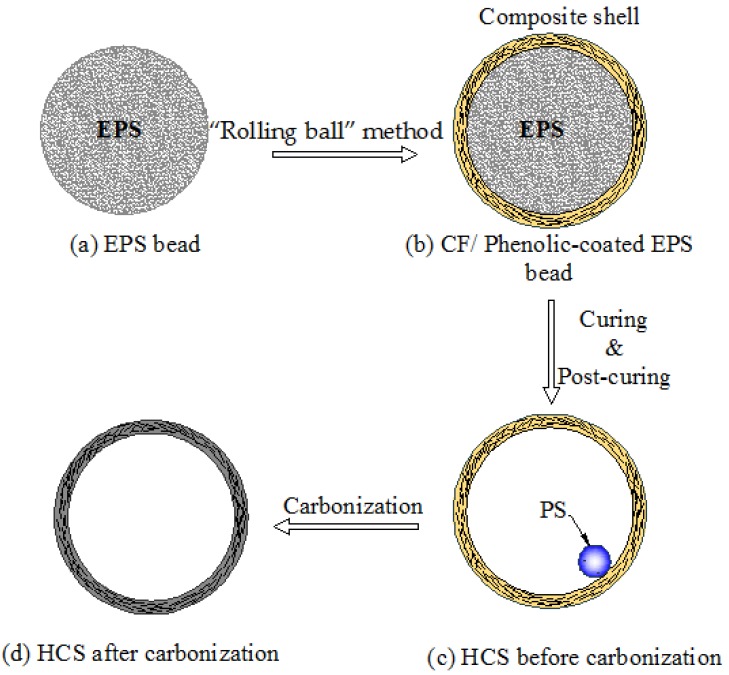
The flow chart illustration of a process for producing hollow carbon spheres (HCSs): (**a**) Expanded polystyrene (EPS) bead; (**b**) Carbon Fiber (CF)/Phenolic-coated EPS bead; (**c**) HCS before carbonization; (**d**) HCS after carbonization.

The manufacture procedures of HCSs were divided into five steps as seen in Figure 1a–d: (a) Cleaning the surface of EPS beads; (b) EPS beads were coated with the layer of carbon fiber/phenolic resin using the “rolling ball” method; (c) The rigid composite shell surrounding the core of the EPS beads was formed after the phenolic resin cured. EPS beads inside the composite shell then shrank after a 1-hour post-cure process at 110 °C; (d) The HCSs were fabricated by carbonization of phenolic resin in a vacuum carbonization device at 1050 °C for 5 h. It should be noted that the carbonization process may be repeated several times in order to reduce the porosities inside the shell.

**Figure 2:**
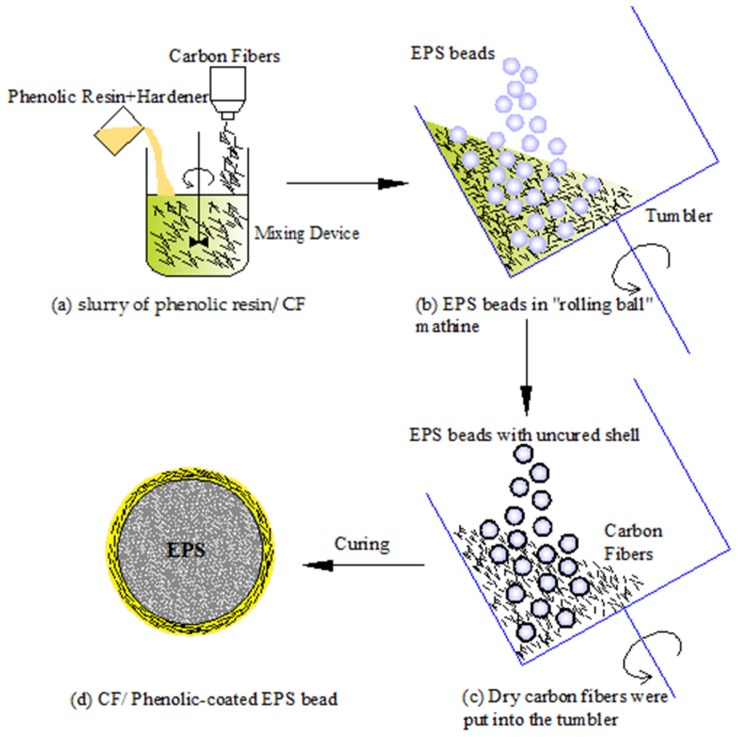
Fabricating procedures by “rolling ball” method: (**a**) slurry of the phenolic resin/CF; (**b**) EPS beads in “rolling ball machine”; (**c**) dry carbon fibers were put into the tumbler; (**d**) CF/Phenolic coated EPS bead.

**Figure 3:**
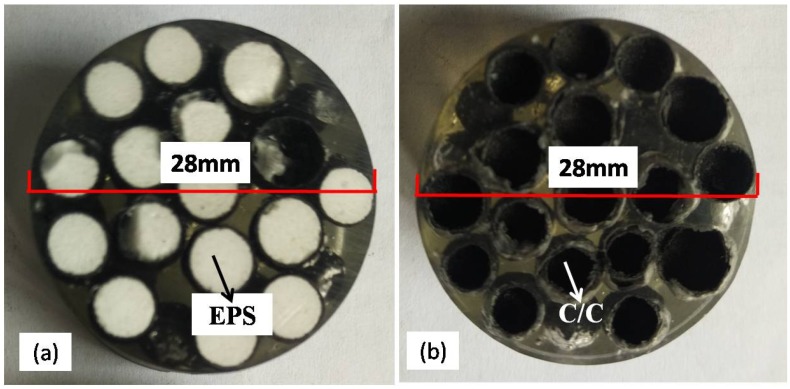
The HCSs (**a**) before carbonization; (**b**) after carbonization.

The “rolling ball” method is the most popular way to prepare HCSs with EPS beads [15,16,17,18]. Figure 2 outlines the manufacture procedures of the experiment. The process can be divided into the following major steps: (1) Preparation of the slurry of carbon fiber/phenolic resin. The phenolic resin and carbon fiber with a volume ratio of 6/4 were put into a container and mixed to form the slurry as shown in Figure 2a; (2) The slurry was moved into a tumbler of the “rolling ball” coater machine with a constant rotation speed of 20–30 rpm. The shell was formed during the process of rotation. Then phenolic resin and carbon fibers were added into the tumbler to form an additional layer until the expected thickness was achieved. The details can be seen in Figure 2b; (3) By the addition of more dry carbon fibers, the uncured shell kept bonding with carbon fibers until it lost tackiness. At this point, the volume content of carbon fibers should be more than 70% of the mixture. The carbon fibers that were not adhered can be reused next time. Finally, all the beads were coated with the maximum volume fraction of carbon fibers as seen in Figure 2c. The process took 30–60 min. The higher the viscosity of the resin, the larger the void defects in the shell; (4) The spheres were cured at 80 °C dwelled for 30 min by blowing hot air while the tumbler was kept rotating. Finally, the core–shell polymer spheres were obtained as seen in Figure 2d.

The low density of HCSs with one iteration of the impregnation–carbonization cycle leads to low compressive strength. Additional impregnation–carbonization cycles should be executed to densify C/C composite shells. More cycles of impregnation-carbonization can reduce the porosity generated by the carbonization of phenolic resin. 

The densification treatment process of the C/C composite shell can be divided into three steps. Firstly, the HCSs were put into a crucible filled with the mixture of phenolic resin and solvent for 10 min in order to permeate the pores inside the C/C composite shell. The spheres were then transferred into the tumbler and kept revolving in order to separate them from each other during the curing process. Finally, the spheres were carbonized in the vacuum carbonization device for another cycle. The carbonization times were decided by the expected density. The shell density increases with the increase of impregnation–carbonization cycles; however, this process consumes more energy. In fact, the density of the C/C composites increased only slightly after four impregnation–carbonization cycles.

### 2.4. Preparation of ASFs

The experimental procedures for fabricating ASF using HCSs were divided into several steps. Firstly, an amount of HCSs was put into a graphite crucible and then gently shaken to improve packing until the spheres were stacked in a maximally dense arrangement. A piece of ceramic mesh was then tailored and installed inside the crucible close to the HCSs. The mesh prevented contact between the melt and the spheres. An ingot of aluminum was put on the mesh and heated at a temperature of 690~720 °C in an electric furnace. The melt was gravity-fed (i.e., it flowed freely, without any application of pressure) into the graphite crucible, resulting in complete infiltration. The graphite crucible was kept slightly vibrating in order to eliminate gas entrapment in the cast samples. The process lasted ca. 20 min. The sample was taken out after natural cooling to room temperature.

## 3. Results and Discussion

### 3.1. Microscopic Analysis before and after the Carbonization

Figure 3 shows images of the cross-section area of HCSs before and after one cycle of carbonization embedded in polymer. The EPS core with the shell of carbon fiber reinforced phenolic resin before carbonization is shown in Figure 3a. The HCSs after carbonization are shown in Figure 3b. The spheres of EPS beads disappeared after carbonization.

**Figure 4:**
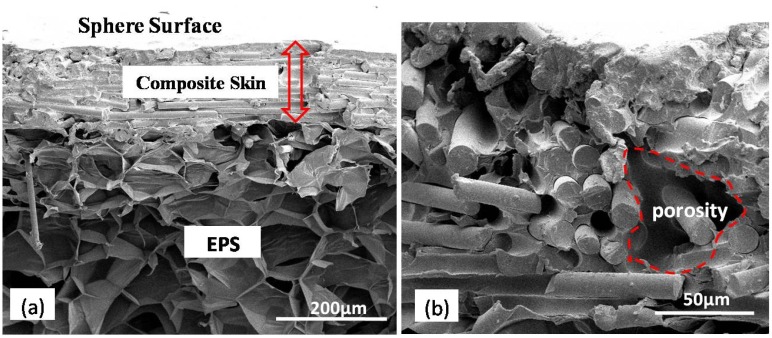
The cross-section appearance of the CF/phenolic resin shell before carbonization: (**a**) Typical structure; (**b**) Magnified image of the CF/phenolic resin composite shell.

Figure 4a shows the typical structure of the EPS core with a shell of CF/phenolic resin before the post-curing process. A large number of air-filled sacs exist in the EPS beads. The thickness of composite shell is approximately 150 μm. Figure 4b is a magnification view of the CF/phenolic resin shell composites. The area inside the dotted line indicates porosity inside the composites. The high viscosity of phenolic resin leads to wrapped air bubbles inside the composite during the process of fabrication. Some air bubbles were also introduced during the stirring process of the mixture with carbon fiber and phenolic resin. Reducing the viscosity of the phenolic resin is beneficial to the reduction of porosity.

**Figure 5:**
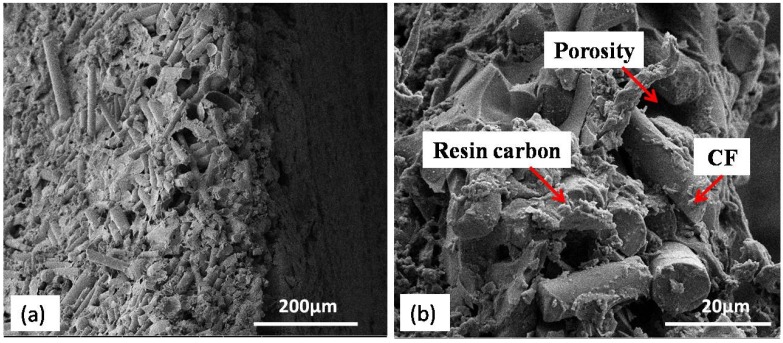
Cross-section of the C/C composite shell: (**a**) Typical structure; (**b**) magnified image.

The micro-structure of the cross-sectional area after carbonization was investigated by SEM as seen in Figure 5a. Compared with the micro-structure before carbonization, the porosity increased significantly. Figure 5b is the magnification view of the shell. The release of gas during carbonization caused increased porosity in C/C composite shells. The carbon yield percentage of the phenolic resin used in this paper is approximately 65%. This means that the cavity volume percentage of the phenolic resin after carbonization can be as high as 35%. There are two approaches generally used to decrease porosity: either to use the precursor with a higher carbon yield percentage or to increase impregnation–carbonization cycles.

### 3.2. Density and Porosity of the HCSs

Fifty HCSs particles before and after carbonization were randomly selected for density testing. The testing results are shown in Table 1. It should be noted that EPS weight in the table after carbonization is the result of calculation. A carbon conversion ratio of 55% was used, following the literature [19]. The average outer diameter of HCS-I was 6.30 mm and the corresponding shell thickness was 0.15 mm. The sizes were dependent on the diameter of the EPS beads and the rolling-ball process. The average outer diameter of HCS-II and corresponding shell thickness were greater than that of HCS-I. This is observed because more impregnation–carbonization cycles lead to adherence of some amount of the resin to the HCSs surface, thus increasing shell thickness.

To calculate the density and void content of the C/C composite shell according to the test results, the following equations were employed:

(1) $$\rho_{\mathrm{shell}}=\frac{M_{\mathrm{HCS}}-M_{\mathrm{EPS}}}{V_{\mathrm{HCS}}-V_{\mathrm{EPS}}}$$

(2) $$V_{\mathrm{voids}}=\left(1-\frac{\rho_{\mathrm{shell}}}{\rho_{\mathrm{shell}}^{\prime }}\right)\times 100\%$$

where $\rho_{\mathrm{shell}}$ is the actual density of composite shell. $M_{\mathrm{HCS}}$ and $M_{\mathrm{EPS}}$ are the weights of the HCS and EPS bead, respectively. $V_{\mathrm{HCS}}$ and $V_{\mathrm{EPS}}$ are the volumes of the HCS and EPS beads, respectively. $V_{\mathrm{voids}}$ is the volume fraction of porosity inside the shell. $\rho_{\mathrm{shell}}^{\prime }$ is the theoretical density of the shell without porosity. The density value of CF/Phenolic resin without porosity is 1.515 g/cm^3^ when the fiber volume fraction is 50%. The density of C/C composite is 1.78 g/cm^3^. The calculation results are listed in Table 2.

It can be seen that the value of $V_{\mathrm{voids}}$ before carbonization is 8.0%. The high viscosity of phenolic resin leads to the high void content. The SEM images also confirmed the existence of porosity. The carbonization of the CF/phenolic resin greatly reduced the density of the composite shell, decreasing the value from 1.394 g/cm^3^ to 1.133 g/cm^3^. Four cycles of repeated phenolic resin impregnation–carbonization contributed to the density increase from 1.133 g/cm^3^ to 1.503 g/cm^3^. Because it is difficult to further impregnate phenolic resin into the shell, the void content value of 15.6% is still at a high level.

### 3.3. Compressive Property of the HCSs

An automatic particle strength tester, KQ-3 (Nanjing Kehuan Analytical instrument Co., Ltd., Nanjing, China), was used to test the compressive properties of the HCSs. Ten particles were randomly selected for the compressive test. The average ultimate failure loads of HCS-I and HCS-II are 24 N and 105 N, with the coefficient of variation of 6.8% and 7.9%, respectively. The reduction of porosity is, therefore, beneficial to the improvement of the compressive strength.

### 3.4. Densities of the ASFs

The following equation is employed to calculate the density of the ASFs:

(3) $$\rho_{\mathrm{ASF}}=\rho_{S}V_{S}+\rho_{A}V_{A}$$

$\rho_{\mathrm{ASF}}$ is the density of the ASF. $\rho_{s}$ and $\rho_{A}$ are the densities of the HCS and aluminum, respectively. The symbols of $V_{S}$ and $V_{A}$ are the volume fraction of the HCSs and aluminum, respectively. The value of $\rho_{A}$ is 2.7 g/cm^3^. The value of $V_{S}$ is 62.0%, which was measured according to ASTM D792-13. The calculated and actual properties of the ASFs are listed in Table 3. Both the two calculated values are higher than the actual values. The existence of a small quantity of fractured HCSs can lead to the deviation.

### 3.5. Macro and Microstructural Characterization of Aluminum ZL101

**Figure 6:**
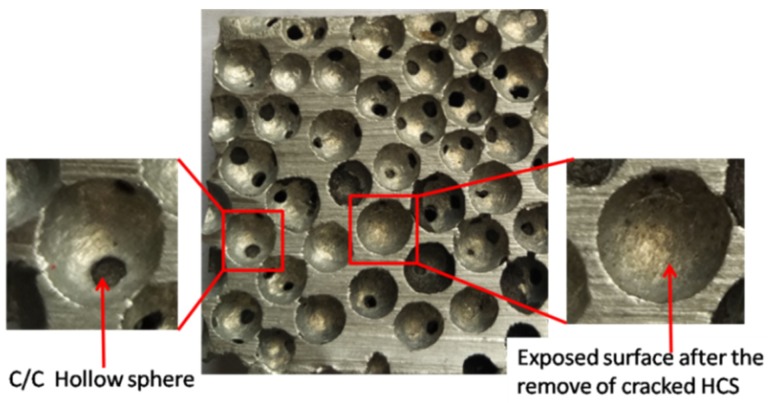
Image of the cross-section showing spherical cavities after the removal of cracked HCSs.

One of the major problems in the fabrication of aluminum-HCSs composites is the poor wettability between HCSs and liquid aluminum [20]. As seen in Figure 6, the smooth surface of the aluminum was exposed inside the spherical cavities after the removal of cracked HCSs. It is important to improve the compressive strength of HCSs in order to achieve high compressive performance of the ASFs.

**Figure 7:**
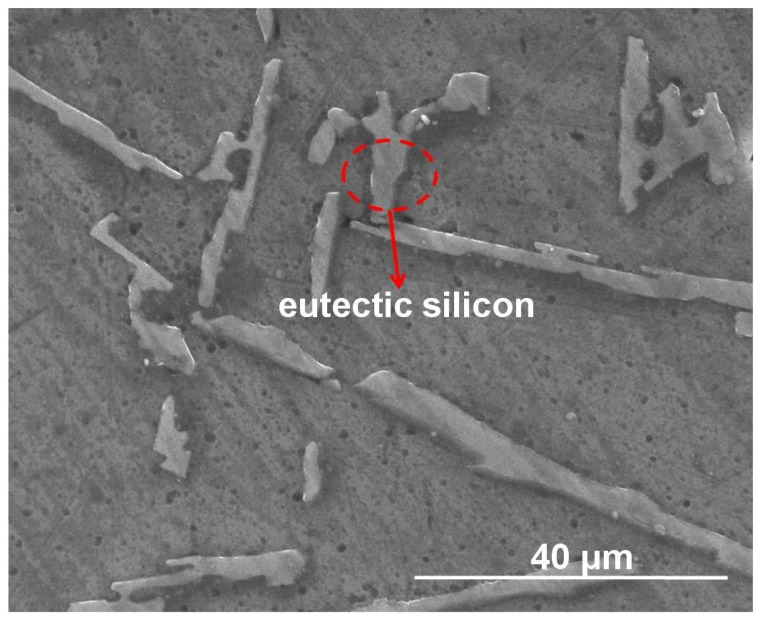
Cross-section morphology of as-cast ZL101 alloy.

**Figure 8:**
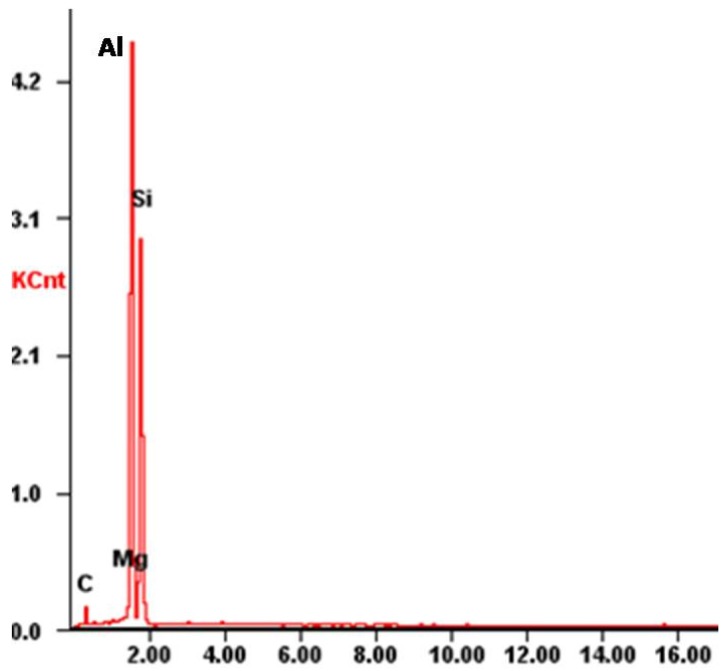
Principal component analysis of eutectic silicon.

Representative morphology of eutectic silicon in ZL101 was observed by SEM, as seen in Figure 7. The particulate and needle-shaped eutectic silicon particles are distributed in the matrix. It also can be seen that there are dispersed tiny holes on the surface, which could decrease the compressive strength of the ASF. Principal component analysis of the eutectic silicon was shown in Figure 8: Al, Si, and Mg. Carbon constitutes the impurities introduced in the sample preparation process.

### 3.6. Compressive Properties and Energy Absorption Capacity of the ASFs

**Figure 9:**
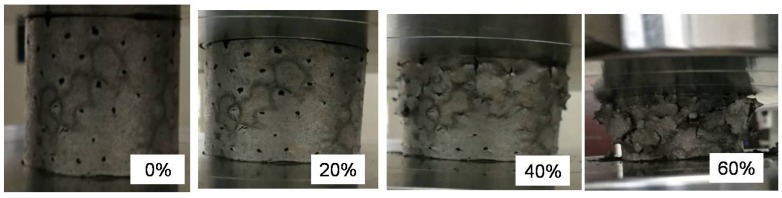
Compression of ASF-II showing different stages of compression: 0%, 20%, 40%, and 60% strain.

**Figure 10:**
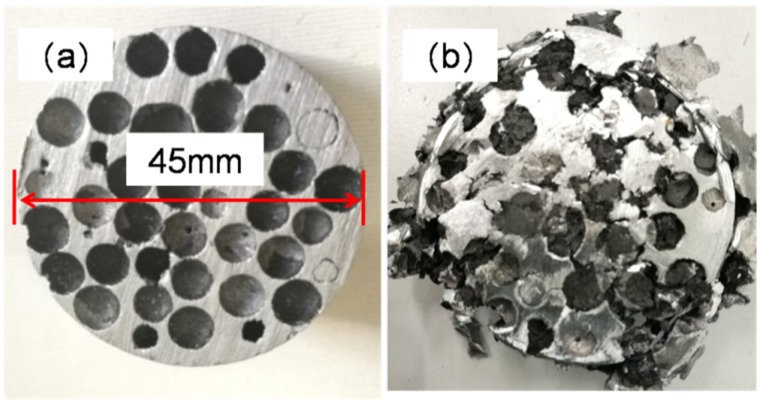
Top views of the ASF-II: (**a**) Before compressive test; (**b**) After compressive test (60% strain).

Insight into the mechanisms of the deformation is provided by images of the ASF-II sample recorded at progressively increasing strains, as shown in Figure 9. The sample deformed smoothly up to 20% strain. The HCSs were completely crushed and some pulverized fragments exfoliated from the sample, as can be seen in Figure 9, at 60% strain. The gaps inside the HCSs were gradually filled by the aluminum matrix during compression. The sample remained intact and in one piece after compression loading. Top views of the foam before and after compressive test are shown in Figure 10.

**Figure 11:**
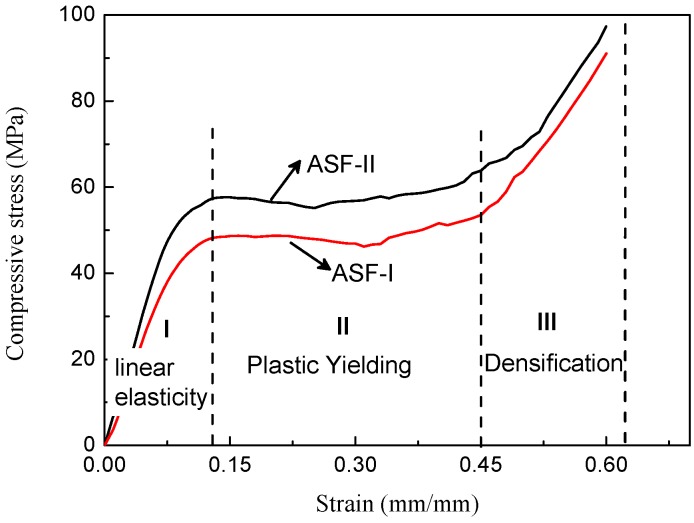
Typical stress–strain curves of the two types of syntactic aluminum foam.

The typical stress–strain curves of the two types of ASF are shown in Figure 11. The compressive test was stopped at 0.6 strain. The ASF produced in this study exhibited a characteristic ductile stress–strain behavior, as seen in the curves. The stress–strain curve could be divided into three stages: the initial stage with a high slope, the second stage with a plateau, and the third stage with a rising slope [21,22]. The initial stage (part I) showed a quasi-elastic deformation. The HCSs embedded in the foam remained intact at this stage. This stage reflects the stiffness of the syntactic aluminum foam and the ability to resist deformation [23]. Peak stress is defined as compressive strength at the end of the initial linear elastic region. The structural stiffness of the ASF is determined by the initial slope of the stress–strain curve [24,25]. The transitional points between initial high slopes and the plateau appear to occur at a strain below 0.15. In the second stage (part II), the plateau region in the stress–strain curves are the result of the collapse of the cells by elastic buckling, plastic collapse, or brittle crushing after initial failure. The plateau region reflects the energy absorption capabilities of the syntactic foam because in the process of deformation a lot of energy is dissipated. In the third stage (part III), a rising slope is related to the densification of the ASF. The densification strain is defined as the strain in the plastic region where stress exceeds the compressive strength value. The stress–strain curve rises until the cavities in the structure completely disappear. The total volume of the syntactic aluminum foam decreases continuously. The ASF-II has higher specific energy absorption capability than ASF-I because of higher compressive strength and plateau strength. These two types of ASF are examples of elastic-plastic materials.

**Figure 12:**
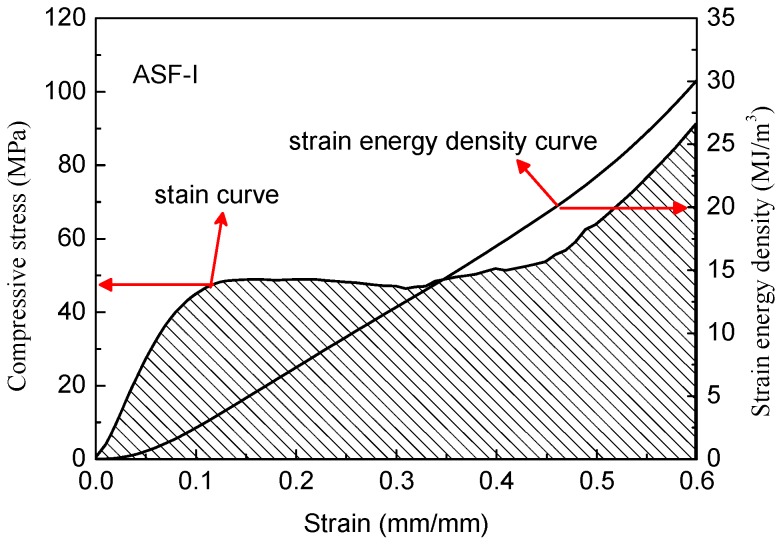
Stress–strain curve and strain energy density curve of ASF-I.

**Figure 13:**
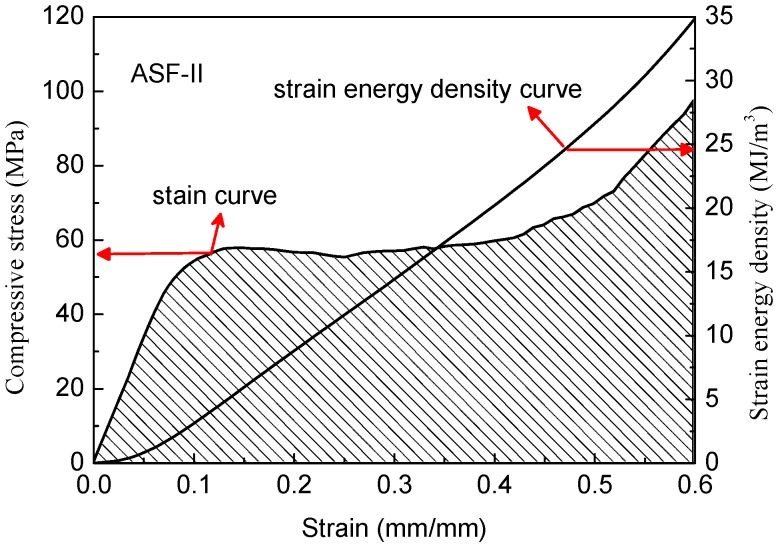
Stress–strain curve and strain energy density curve of ASF-II.

As shown in Figure 11, the ASFs exhibit a long plateau in the stress–strain curve which allows energy absorption at a nearly constant load. The absorbed specific energy is equal to the area under the recorded stress–strain curve and can be integrated numerically [26]. According to Formula (3), curves were drawn by integrating the stress–strain curves as shown in Figure 12 and Figure 13.

(4) $$W=\int_{0}^{\varepsilon }\sigma d\varepsilon$$

where W is the energy absorption capacity per unit volume, σ is the stress, and ε is the strain. 

All the ASFs produced in this study have great energy absorption capacity. The ASF-I absorbs on average 30.1 MJ/m^3^ with a deviation of 6.7% (26.4 KJ/Kg), while ASF-II absorbs on average 34.9 MJ/m^3^ with a deviation of 9.2% (28.8 KJ/Kg). The energy absorption capacity of ASF-II increased by 15.8% per cubic meter and 9.1% per kilogram compared with ASF-I. The consumed mechanical energy increased significantly by improving the compressive strength of the HCSs as a filler material. It is easy to understand that the reduction of defects in the shell of HCS-II leads to the increase of the energy absorption capacity of ASF-II. 

Table 4 shows the comparison of energy absorption capacity of our samples with other ASFs in the published literature [3,4,27,28,29,30]. All the energy absorption values were obtained from stress–strain curves. The low density closed cell aluminum foams without any particles had lower energy densities than the ASFs filled with particles. The energy absorption values of ASFs filled with expanded perlite particles and ceramic cenospheres W125 was lower than ASF-I and ASF-II. The ASF filled with hollow ceramic microspheres has better capacity than ASF-I and ASF-II, as seen in the table. However, the hollow ceramic microspheres were hard to infiltrate with molten aluminum because of their small size. Air pressure had to be applied in order to force the molten aluminum into the spaces between the hollow microspheres. This method increased the complexity of the process and its cost.

## 4. Conclusions

In this paper, core–shell polymer spheres were produced with the “rolling ball” method using EPS beads as sacrificial templates. The HCSs were successfully fabricated by carbonizing the core–shell polymer spheres. The shell material was made of carbon fiber/phenolic resin. Based on the test results, increasing the number of impregnation–carbonization cycles could effectively reduce the porosity of the C/C composite shell to produce HCSs with more flawless microstructure. The C/C shell density increased from 1.133 g/cm^3^ to 1.503 g/cm^3^. The sphere density increased from 0.16 g/cm^3^ to 0.25 g/cm^3^. The ultimate failure load of the HCSs increased from 24 N to 105 N. The strain energy density of the corresponding ASFs manufactured by melt infiltration increased from 30.1 MJ/m^3^ to 34.9 MJ/m^3^ at the strain value of 0.6. The results indicated that the ASFs in this paper had good energy absorption capacity.

The HCSs studied in this paper have lower density than many other hollow spheres popularly used in metal matrix syntactic foams. It also provides a new choice for the preparation of low density metal matrix syntactic foams by packing the macro-particles of HCSs with hollow microspheres, like ceramic hollow spheres. The ASFs exhibit a potential use in the field of energy absorption applications.

## Figures and Tables

**Table 1:** Parameters of HCSs before and after carbonization: Size and density.

Name	Carbonization Times	Particles	HCSs Weight (g)	EPS Weight (g)	Outer Diameter (mm)	Shell Thickness (mm)	HCS Density (g/cm^3^)
/	0	50	1.2988	0.0011	6.30 ± 1.15	0.15 ± 0.01	0.198
HCS-I	1	50	1.0403	0.0006	6.30 ± 1.15	0.15 ± 0.01	0.159
HCS-II	4	50	1.6532	0.0006	6.36 ± 1.15	0.18 ± 0.02	0.245

**Table 2:** Parameters of HCSs before and after carbonization: Shell density and volume fraction of porosity.

Name	Times of Carbonization	Shell Density (g/cm^3^)	Volume Fraction of Porosity (%)
/	0	1.394	8.0
HCS-I	1	1.133	36.3
HCS-II	4	1.503	15.6

**Table 3:** The calculated value and actual value of the ASFs.

Name	HCS Type	Calculated Density (g/cm^3^)	Actual Density (g/cm^3^)
ASF-I	HCS-I	1.12	1.14
ASF-II	HCS-II	1.18	1.21

**Table 4:** Comparison of energy absorption capacity of our samples with other ASFs.

Foam	ASF-I	ASF-II	Closed Cell Aluminum Foam [28]	ASF	ASF	ASF
Hollow sphere	C/C	C/C	No	expanded perlite particles	ceramic cenospheres W125	hollow ceramic microsphere
Sphere density (g/cm^3^)	0.16	0.25	/	0.18	0.7	0.6–0.8
Diameter	6.30 mm	6.36 mm	/	irregular shapeca. 4 mm	75 μm	15–75 μm
Aluminum matrix	Al alloy ZL101	Al alloy ZL101	multiple types Al alloy	Alalloy A356	commercial pure Al	commercial-purity Al and 7075-T6 Al alloy
Densification strain	0.6	0.6	0.6	0.5	0.5	0.55–0.6
Foam density (g/cm^3^)	1.14	1.21	0.2–0.6	1.02–1.06	2.2–2.4	1.4–1.66
Energy absorption per volume at densification (MJ/m^3^)	30.1	34.9	1–10	11.9–23.6	26.0–34.9	55 (cp-Al, strain: 0.6) 80 (7075-T6, strain: 0.55)
Energy absorption per mass at densification (KJ/Kg)	26.4	28.8	/	11.3–22.3	11.6–12.7	39.3 (cp-Al) 48.2 (Alloy T6)
